# Can Epigenetics of Endothelial Dysfunction Represent the Key to Precision Medicine in Type 2 Diabetes Mellitus?

**DOI:** 10.3390/ijms20122949

**Published:** 2019-06-17

**Authors:** Celeste Coco, Luca Sgarra, Maria Assunta Potenza, Carmela Nacci, Barbara Pasculli, Raffaela Barbano, Paola Parrella, Monica Montagnani

**Affiliations:** 1 Department of Biomedical Science and Human Oncology, University of Bari “Aldo Moro”, 70124 Bari, Italy; cococeleste@libero.it (C.C.); sgarraluca@gmail.com (L.S.); mariaassunta.potenza@uniba.it (M.A.P.); carmela.nacci@uniba.it (C.N.); monica.montagnani@uniba.it (M.M.); 2 Laboratory of Oncology, Fondazione IRCCS Casa Sollievo della Sofferenza, 71013 San Giovanni Rotondo (Foggia), Italy; b.pasculli@operapadrepio.it (B.P.); r.barbano@operapadrepio.it (R.B.)

**Keywords:** epigenetic changes, type 2 diabetes, endothelial dysfunction, antidiabetic drugs

## Abstract

In both developing and industrialized Countries, the growing prevalence of Type 2 Diabetes Mellitus (T2DM) and the severity of its related complications make T2DM one of the most challenging metabolic diseases worldwide. The close relationship between genetic and environmental factors suggests that eating habits and unhealthy lifestyles may significantly affect metabolic pathways, resulting in dynamic modifications of chromatin-associated proteins and homeostatic transcriptional responses involved in the progression of T2DM. Epigenetic mechanisms may be implicated in the complex processes linking environmental factors to genetic predisposition to metabolic disturbances, leading to obesity and type 2 diabetes mellitus (T2DM). Endothelial dysfunction represents an earlier marker and an important player in the development of this disease. Dysregulation of the endothelial ability to produce and release vasoactive mediators is recognized as the initial feature of impaired vascular activity under obesity and other insulin resistance conditions and undoubtedly concurs to the accelerated progression of atherosclerotic lesions and overall cardiovascular risk in T2DM patients. This review aims to summarize the most current knowledge regarding the involvement of epigenetic changes associated with endothelial dysfunction in T2DM, in order to identify potential targets that might contribute to pursuing “precision medicine” in the context of diabetic illness.

## 1. Introduction

The growing prevalence of type 2 diabetes mellitus (T2DM) worldwide and the increased burden in terms of social and economic costs, health resources used, and lost productivity associated with T2DM underline the need to identify novel biomarkers with high specificity and sensitivity for early-stage diabetic patients, with the purpose of fostering strategies to prevent diabetes and associated complications.

### 1.1. The Potential Predictive Role of Endothelial Dysfunction in T2DM Cardiovascular Risk

T2DM is a multifactorial chronic metabolic disease resulting from a complex interaction between environmental factors and genetic background. This concept implies that the early identification of individuals at risk for T2DM is extremely important to personalize the therapeutic management of each patient, in the attempt to limit the progression of the disease and prevent morbidity and mortality risk. Although genome-wide association studies (GWAS) found a number of genes involved in susceptibility to T2DM, genetic testing cannot accurately predict the clinical risk and/or pathological complications of diabetic patients [1,2]. On the other hand, recent data suggest that epigenetic mechanisms, such as DNA methylation, changes of chromatin through post-translational histone-modification and non-coding RNAs may represent a crucial interface between genetic predisposition and environmental factors [3,4,5] and play a key role in the pathogenesis and progression of T2DM complications.

It is commonly recognized that T2DM is an independent risk factor for cardiovascular diseases (CVD). In the natural history of diabetes, multiple mechanisms contribute to cardiovascular damage. With the growing understanding of the functional role played by the endothelium and the subsequent discovery of several endothelial mediators and their respective mechanism of action, it has become commonly accepted that endothelial abnormalities represent an early sign of metabolic disturbances [6,7,8,9]. In turn, each of the metabolic derangements occurring in diabetes (insulin resistance and compensatory hyperinsulinemia, hyperglycaemia, oxidative stress, excess free fatty acid release and lipotoxicity) may impact on endothelial function individually and contribute to reinforcing the negative activity of all other players [10,11,12,13]. The resulting effects on perturbation/injury of the endothelial permeability, adhesion and transmigration of monocytes/macrophages into the intima, foam cell formation, migration and proliferation of medial vascular smooth muscle cells concur to increase intima hyperplasia, enhanced coagulability and impaired fibrinolysis, in the end promoting vascular occlusion and increasing the risk of cardiovascular events in T2DM patients. Moreover, the notion that endothelial changes are, to a certain extent, a reversible process implies that evaluation of endothelial function over time may be useful to assess the efficacy of individual treatments. Therefore, in the last years, the search for reliable biomarkers assessing endothelial activity has become increasingly relevant. 

### 1.2. Endothelial Dysfunction Under Diabetes

The main characteristics of endothelial activity and the specifics of the insulin signalling pathway related to the synthesis and release of endothelial mediators have been deeply investigated and described elsewhere [11,14]. Endothelial dysfunction, the earliest marker of vascular alteration, is a condition resulting from the impaired bioavailability of the gaseous mediator nitric oxide (NO). The physical nature of this molecule and its short half-life require the perfect assembly of a complex machinery that produces NO when needed. NO is generated from the conversion of the amino acid L-arginine by the endothelial NO synthase (eNOS), which can be activated by Ca++-dependent pathways and by a variety of kinases including 5’AMP-activated protein kinase (AMPK) and PKB/Akt. In the absence of adequate levels of L-arginine or insufficient amounts of co-factors FAD, NADPH and tetrahydrobiopterin (BH4), eNOS may become uncoupled and generate oxygen free radicals (ROS) instead of nitrogen species.

Under T2DM, both insulin resistance and compensatory hyperinsulinemia may compromise endothelial function by causing imbalance in the expression/activity of eNOS, by reducing the amounts of eNOS substrates/cofactors, and/or by accelerating the conversion of NO into ROS. The consequences consist of reduced vascular relaxation, increased expression of adhesion molecules such as VCAM1 and E-selectin and a stronger predisposition to pro-atherogenic and inflammatory events [15]. 

Later in the disease progression, overt hyperglycaemia fosters the non-enzymatic formation of advanced glycosylated end products (AGEs) and may activate matrix-degrading metalloproteinases, enzymes implicated in plaque rupture and arterial remodelling [16,17,18]. Importantly, early and long-lasting exposure to hyperglycaemia can leave an imprint in vascular cells and alter the expression of genes in endothelial, smooth muscle, retinal and cardiac cells, without changes on the DNA sequence: chronic hyperglycaemia may induce the AGEs-mediated glycation of mitochondrial proteins that lead to a decline in mitochondrial function and an excessive production of ROS [19,20,21]. The mitochondrial respiratory chain proteins that undergo glycation become more prone to produce superoxide anion, regardless of the level of hyperglycaemia, damaging mitochondrial DNA (mtDNA), which is considered even more sensitive to oxidative damage than nuclear DNA is [22,23,24,25]. The long-term persistence of these epigenetic abnormalities, which may become irreversible, represents the key mechanism underlying the phenomenon of “metabolic memory” [26], which refers to an altered gene expression responsible for the progression of the most feared micro- and macro-vascular diabetic complications, even when the levels of glycaemia are normalized [27,28].

### 1.3. The Phenomenon of Metabolic Memory in Endothelium

The concept of “metabolic memory” arises from experimental and clinical observations reporting that early exposure to a hyperglycaemic environment is “recorded” by the cells and may contribute to explain vascular complications observed in diabetic patients whose glycaemic control has not been achieved very early [27,28,29]. Since the molecular changes concern mainly the endothelium, the most appropriate expression for this phenomenon would be the “endothelial hyperglycaemic memory.”

The notion that the damage induced by hyperglycaemia can be limited or prevented when glycaemic control is achieved early but not easily reversed if poor control lasts longer, implies that high glucose levels may trigger a variety of changes that persist for days after the normalization of glucose levels. Initially, hyperglycaemia, increased oxidative stress and excessive AGE formation are linearly associated. Later, a persistent respiratory chain protein glycation and mitochondria DNA damage could generate a hyperglycaemia-independent vicious cycle [27] in which oxidative stress is self-supporting and inflammatory processes (induced by receptor binding of AGEs or oxidative stress) modify the composition and structure of the extracellular matrix [28] with subsequent fibrosis and interference with capillary density and blood flow capacity. Epigenetic mechanisms have been hypothesized to be a critical interface between genetic and environmental factors to explain metabolic memory and will be discussed in the following paragraphs.

While the progressive understanding of the molecular mechanisms regulating insulin activity in endothelium has suggested crucial targets for novel therapies aimed at preventing diabetes-related vascular complications [6], not all the current antidiabetic drugs target endothelial cells (EC) in the same way or have the same protective potential [30]. Concomitantly, although hyperglycaemia-mediated cardiovascular impairment can be controlled pharmacologically through standard therapies (combined treatment with hypoglycaemic, antihypertensive and anti-inflammatory drugs), dietary modifications and exercise, several patients continue to develop life-threatening vascular complications for which current therapies have not been fully effective [30].

**Figure 1:**
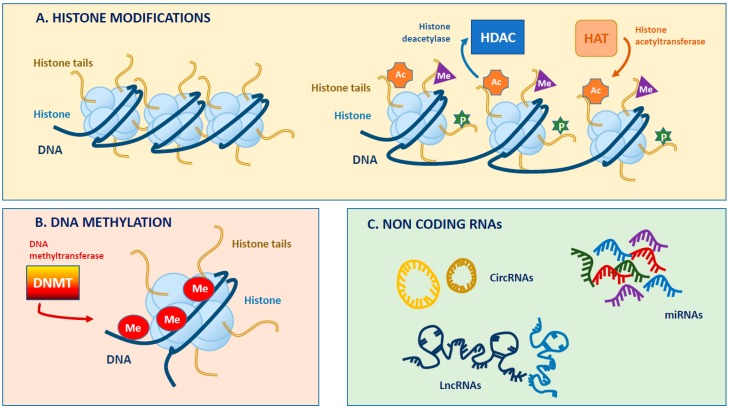
Simplified overview of main epigenetic modifications. (**A**). Chromosomal DNA is packaged around histone proteins to form nucleosomes. Nucleosome opening and accessibility to nuclear factors is regulated, in part, by post-translational modifications of histone tails that include phosphorylation, ubiquitination, acetylation and methylation. Acetylation reactions on lysine (K) residues by histone acetyl-transferase (HAT) mask the positive charges of the aminoacidic side chains and thus allow chromatin to assume a less condensed conformation permissive for transcription. In contrast, deacetylation reactions by histone deacetylase (HDAC), increasing the chromatin packaging, prevent DNA transcription. (**B**). DNA methylation consists of adding a methyl group to the carbon-5 position of a cytosine in the so-called CG Islands by DNA-methyltransferase (DNMT) enzymes. The methylation of CpG islands may induce gene silencing by preventing the binding of transcription factors (TFs) to the promoter or favouring the binding of proteins endowed with a methylated CpG (MeCpG Binding-Protein). DNA methylation promotes the persistence of certain histone states, such as deacetylation, thus providing a mechanism for perpetuating post-translational histone modifications. (**C**). Non-coding (nc) RNAs are conventionally grouped into small ncRNAs and long ncRNAs. According to their function, ncRNAs can be classified in constitutively expressed housekeeping molecules and regulatory molecules such as micro (miRNA) and long non coding (lncRNAs) RNAs. Among these molecules, miRNAs are the most extensively studied and represent the principal epigenetic regulators of gene expression, acting predominantly at the post-transcriptional level.

In this intricate scenario, the occurrence of inter-individual differences in drug response has highlighted the potential role of genetic polymorphisms in affecting absorption, bioavailability, efficacy and safety of anti-diabetic therapies. The presence of most single-nucleotide polymorphisms (SNPs) associated with diabetic disease in non-coding regions of the genome or in other regulatory regions such as enhancers can influence gene expression by altering the binding of transcription factors [1]. For these reasons, the evaluation of epigenotypes by epigenome-wide association studies (EWAS) is expected to provide new critical information on the pathogenesis of diabetic complications and metabolic memory as well as help identify new therapeutic modalities and diagnostic biomarkers for early intervention. Epigenetic studies of endothelial dysfunction associated with diabetes might undoubtedly represent a promising strategy for recognizing subjects with a greater susceptibility to developing micro- and macrovascular complications; concomitantly, pharmacogenetics and pharmacogenomics knowledge including epi-biomarkers might set the basis for a new therapeutic approach, that selects for each diabetic patient the more appropriate therapy to increase the survival with a lower risk of toxicity [3]. In few words, epigenetic traits may represent new targets for individualized therapy and pave the way for precision medicine in diabetes. The following paragraphs recapitulate the main epigenetic mechanisms that regulate gene expression, focusing on DNA methylation, histone modifications, chromatin remodelling and non-coding RNAs (see also [31]) (Figure 1).

## 2. What Epigenetics Really Means

Regulation of gene expression is a composite and multifaceted process. Gene sequences harbour various positive regulatory elements, including a promoter located immediately upstream of the transcription start site (TSS) and several enhancers that may be located farther away (upstream of the TSS, downstream of the gene or within an intron). Enhancers represent the docking points for many transcription factors that are DNA-binding proteins in sequence-specific way [32]. Then, the transcription factors recruit the “cofactors,” proteins that indirectly modulate gene expression by modifying the activity of a bound transcription factor. In general, cofactors are defined co-activators or co-repressors when they regulate gene expression positively or negatively, respectively. Thus, a transcriptional event reflects the net balance between co-activators and co-repressors bound to DNA at a given time; this dynamic process can be regulated by the activation of intracellular signalling pathways and nutritional status [32].

Epigenetic encompasses all modifications functionally relevant into the nuclear chromatin that change gene expression but that are not caused by alterations (mutations) in the primary DNA sequence [33,34]. These alterations, representing the epigenome, can be either inherited or accumulated throughout a lifetime and have effects on the cellular repertoire of active genes [35]. The epigenomic code is dynamic and it is responsible for some inheritable differences in phenotype that occur when the DNA code is stable or unchanged, including for example continuous modifications of chromatin function due to aging [36] or to changes in nutrition and fluctuations in metabolism [37]. Many epigenetic variations, responsible for significant differences in gene expression within the same species, do not involve coding genes but occur in non-coding regions of the genome [38,39]. The epigenome may represent the interface between genetic variants and environment, as epigenetic alterations undergo dynamic changes during development and in response to nutritional, behavioural and environmental stimuli [33]. 

### 2.1. DNA Methylation

DNA methylation that occurs symmetrically on the two DNA strands consists of adding a methyl group to the carbon-5 position of a cytosine (C) in the CG stretches by DNA-methyltransferase (DNMT) enzymes. The so-called CG Islands (or CpG Islands) are repeated CG sequences, 500 to 2000bp long, usually located close to or within the gene promoters, that tend to be unmethylated in the housekeeping genes and in tissue-specific genes for allowing transcription but hypermethylated in the genes not necessary for that cell line, thus hypomethylated gene promoters are more active compared to hypermethylated genes [40]. The methylation of CpG islands may induce gene silencing by preventing the binding of transcription factors (TF) to the promoter or favouring the binding of proteins endowed with a methylated CpG (MeCpG Binding-Protein) and the recruitment of chromatin modifying enzymes [41,42]. There are three different DNMT enzymes catalysing the DNA methylation: DNMT3a and DNMT3b are closely related and involved in de novo methylation on both strands. On the other hand, DNMT1 maintains methylation status during replication, recognizing the mCpG dinucleotide on the mother strand and the methyl nucleotide (CpG GpCm) on the child strand, providing the conservation and heritability of methylated sites in the cell line [43,44]. However, DNA methylation alone is not sufficient to abolish gene expression and other epigenetic processes, such as deacetylation and methylation of histones are necessary.

### 2.2. Histone Modifications

The histone deacetylase (HDAC) and histone methyltransferase (HMT) are enzymes that start the chromatin condensation, preventing activation of transcriptional machinery [40,45]. Histones are nuclear proteins closely DNA-associated and play an important role in the regulation of gene expression. Five types of histones have been identified: H1 (or H5), H2A, H2B, H3, H4. Histone-protein 1 and its homologous 5 (H1, H5) are involved in superior chromatin structures; the other types of histones are associated to nucleosomes [46]. Post-translational modifications of histones, which include methylation, acetylation, ubiquitination and phosphorylation, may cluster in different patterns to regulate chromatin architecture [47] and represent the components of epigenetic transcription regulation most studied. These reactions occur more frequently on the tails of H3 and H4 histones and their impact on gene expression can be different depending on the specific chemical modifications. Acetylation and methylation are the chemical modifications better investigated but only partially clarified so far [48]. Acetylation reactions on lysine (K) residues by histone acetyl-transferase (HAT) mask the positive charges of the aminoacidic side chains and thus allow chromatin to assume a less condensed conformation permissive for transcription. In contrast, deacetylation reactions by histone deacetylase (HDAC), increasing the chromatin packaging, prevent DNA transcription [49]. The acetylation reaction is an active marker on chromatin, is reversible and does not necessarily have mitotic heritability [46]. Histone lysine (K) and arginine (R) methylation is a posttranslational reversible modification performed by specific enzymes such as lysine methyltransferase (KMT) [50]. The amino acids can be mono-, di- or tri-methylated and they function as anchorage sites for other non-histone proteins that bind methyl groups. The functional role of histone methylation on gene expression changes according to the histone, the methylated amino acid residue and the number of methyl units added. For example, lysine mono-methylation of histones is generally associated with gene repression, while di- or tri-methylation can enhance gene transcription (e.g., H3K4me3) and induce either gene silencing (H3K9me3/me2) [48] or gene repression associated with hypermethylation of the CpG islands within the promoter (H3K27me3) [46]. 

### 2.3. Chromatin Remodeling and Non-coding RNAs

Although ncRNAs do not directly affect chromatin architecture, they play an essential role in post-transcriptional regulation of gene expression. Based on their size, they are conventionally grouped into two major categories: small ncRNAs (<200 nt) and long ncRNAs (lncRNAs >200 bp). According to their function, ncRNAs can be classified in constitutively expressed housekeeping molecules, such as ribosomal (rRNA), transfer (tRNA), small nuclear (snRNA) and small nucleolar (snoRNA) RNAs and regulatory molecules such as small interfering (siRNAs), piwi-associated (piRNAs), micro (miRNA, miRs) [51,52] and long non coding (lncRNAs) RNAs [53]. Among these molecules, miRNAs are the most extensively studied and represent the principal epigenetic regulators of gene expression, acting predominantly at the post-transcriptional level [54,55,56].

MicroRNAs (miRNA) are small (18–25 nucleotides), highly-conserved non coding RNAs, which induce mRNA degradation and/or inhibit translation of protein-coding genes by binding to the 3′untranslated regions (UTR) of target mRNAs, via a short complementary sequence of 6–8 nucleotides, known as seed sequence [57]. In order to repress the transcript, it is crucial that the seed sequence recognizes almost perfectly the regions at the 3′UTR of their target genes [58]. Furthermore, miRNAs could also bind to complementary sequences present in the promoter, competing for transcription factor binding sites. It seems that a single miRNA could modulate expression of several hundred genes, considering that more than half of protein-coding genes carry miRNA target sites in their 3′UTR regions [59,60]. The promoters of almost the 50% of the miRNA genes are embedded in CpG islands, suggesting that miRNA transcription can be epigenetically regulated [61].

LncRNAs are long transcripts similar to messenger RNAs, including long intergenic ncRNAs (lincRNA), enhancer ncRNAs (eRNAs), natural antisense transcripts (NATs) and others [62]. As mRNAs, they are frequently transcribed by RNA polymerase II and then subjected to post-transcriptional, also epigenetic, modifications but lack protein-coding (translational) potentials. LncRNAs exhibit tissue-specific expression and are able to fold into complex secondary or tertiary structures interacting with different types of molecules and attending to multiple regulatory networks, including chromatin remodelling, alternative splicing, transcriptional and post-transcriptional gene regulation [62].

## 3. Epigenetic Changes in T2DM-Related Endothelial Dysfunction

As mentioned before, the pandemic dimension of T2DM and associated social and economic burden strengthens the search of additional biomarkers that may help to identify early-stage alterations of diabetes. Currently, the risk to develop T2DM -as well as the disease progression in T2DM patients- is evaluated according to serum parameters (including glucose levels, HbA1c, triglycerides, cholesterol, lipoproteins, C peptide), anthropometric characteristics (body mass index (BMI), waist circumference, blood pressure, sex) and lifestyle habits (including unhealthy eating habits, lack of exercise, smoking). Alongside these classic biomeasures for T2DM, several inflammatory markers—such as serum levels of adipokines, cytokines, the high-sensitive C-reactive protein (hs-CRP) and imaging techniques—are widely used; unfortunately, especially at an early stage of the disease, none of these measurements alone accurately predicts the risk of diabetes and its complications [63]. In the attempt to fill this gap, new molecules, including alpha-hydroxybutyric acid (AHBA), linoleonylglycerophosphorcholine (LGPC) and oleic acid, are emerging as potentially useful biomarkers to detect initial insulin resistance in subjects at high risk of T2DM, some years before the clinical onset of the disease (Quantose IR test) [64,65]. 

In this perspective, the growing knowledge of epigenetic mechanisms involved in metabolic memory may represent the turning point to identify novel risk biomarkers. Some recent evidences suggest that factors acting during prenatal life, such as malnutrition or stress, can induce epigenetic changes in different tissues and organs, increasing the risk of coronary heart disease and T2DM in old age [66]; similarly, epigenetic modifications in foetal life may modify a series of parameters such as secretion and sensitivity to insulin, production and synthesis of hepatic glucose, release of hormones involved in glucose metabolism, contributing to the increased risk of developing T2DM in adulthood [29]. It is also accepted that epigenetic mechanisms could be related to the maintenance of inflammation, contributing to the progression of diabetes and its vascular complications [67]: the inflammatory phenotype of cells involved in immune responses, including endothelial cells (ECs), triggers the activation of the NF-kB nuclear complex that induces the transcription of a number of genes related to the inflammatory response, such as those coding for both cytokines (IL1, IL6 and TNFα) and adhesion molecules (VCAM1, ICAM1 and MCP-1). These genes are activated chronically in endothelial and peripheral blood cells exposed to transient hyperglycaemia or obtained from diabetic patients [68,69,70] and are involved in the progression of diabetic vascular complications such as atherosclerosis and retinopathy [2]. Epigenetic modifications in the NF-kB promoter region produce an increased expression of p65 subunit of NF-kB, with subsequent increased activation of NF-kB pathway [70,71,72]. 

### 3.1. DNA Methylation and Histone Modifications

Post-translational histone modifications (PTHM) and DNA methylation are the most abundant epigenetic modifications involved in metabolic memory, responsible for long-lasting chromatin remodelling and vascular epigenetic changes that cause persistent increase in proatherogenic gene expression even after the restoration of normoglycemic conditions [73]. In human aortic endothelial cells (HAEC), histone methyltransferase Set7 plays a key role for sustained vascular gene expression in response to previous hyperglycaemia [73,74]. Indeed, Set7 gene-silencing abolishes the NF-kB-dependent oxidative and inflammatory signalling pathway, suggesting that the inhibitors of Set7 could be designed to erase metabolic memory and to avoid diabetic vascular complications [74,75]. 

**Figure 2:**
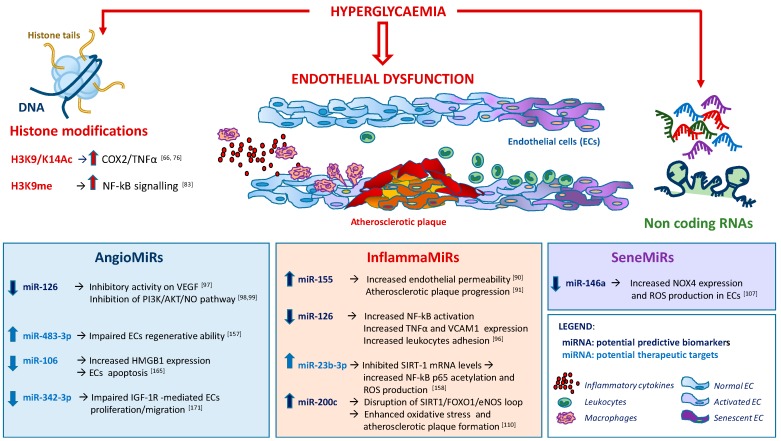
Epigenetic changes in T2DM-related endothelial dysfunction. Schematic examples of hyperglycaemia-associated histone modifications in endothelium, with subsequent upregulated expression of pro-inflammatory signalling pathways and representative angiogenesis-associated miRNAs (angioMiRs), inflammation-associated miRNAs (inflammaMiRs) and senescence-associated miRNAs (seneMiRs) whose levels are deregulated under diabetes. Advances in understanding the mechanisms involved in reversible epigenetic changes will hopefully help to identify epigenetic biomarkers and novel therapeutic targets for additional interventions that can delay the harmful effects of diabetes on endothelial function.

A close correspondence between gene-activating histone acetylations and the expression of proinflammatory molecules under diabetes is supported by the over-expression of TNFα and COX-2 genes in blood monocytes from diabetic patients, which has been related to the hyperacetylation of H3K9/K14 histone [29,76]. Consistent with this hypothesis, hyperacetylation of H3K9/K14 histone and subsequent up-regulation of genes involved in metabolic and cardiovascular diseases has been observed in primary vascular endothelial cells exposed to hyperglycaemia [77] (Figure 2).

In human diabetic retinopathy, the histone hypomethylation of H3K4 is accompanied by a down-regulation of gene expression for the antioxidant enzyme superoxide dismutase 2 (SOD2) [78]. Concomitantly, an increased activity of matrix metalloproteinases (MMPs) has been observed in patients with diabetic retinopathy as well as in the retina of diabetic mice [79,80]. MPP-9 activation in the retina is considered one of the key events that damage mitochondrial function and activate the apoptotic machinery [81,82]; according to mechanistic interpretation, diabetes causes epigenetic changes on MPP-9 secondary to H3K9 hypomethylation and increased acetylation on lysine (K9) at the MPP-9 promoter. Acetylation on H3K9 augments chromatin accessibility and induces NF-kB recruitment which in turn activates MPP-9 and accelerates mitochondrial damage and capillary cell apoptosis in diabetic patients [83]. Taken together, all these findings support the direct role of hyperglycaemia in histone post-translational regulation, highlighting the need to further understand how to reverse epigenetic modifications to prevent progression of vascular complications despite glycaemic control.

Other epigenetic changes, including the DNA hypomethylation at the LINE-1 sequences (Long Interspersed Nuclear Element 1), may be associated with cardiovascular and metabolic complications in T2DM. LINE-1 sequences are highly repeated human DNA sequences that constitute about 17% of the human genome [84] and the degree of LINE-1 DNA methylation may provide a link between environmental exposure and development of cardiovascular complications of T2DM independently of other classical risk factors [85]. Indeed, higher levels of LINE1 methylation have been associated to a reduction in cholesterol/HDL cholesterol ratio and considered predictive of less weight gain over time and subsequent lower body mass index (BMI) in female patients [86].

### 3.2. Chromatin Remodeling and Non-coding RNAs

In the search for new biomarkers able to detect pre-symptomatic subjects or individuals at high risk of developing diabetes, many efforts have been devoted to understanding the regulatory function of miRNAs and lncRNAs ECs, where their deregulation may correlate with the risk of developing vascular complications. In the human genome, 2654 miRNAs control more than 60% of the protein-coding genes [87,88]. With respect to mechanisms implicated in EC function/dysfunction associated to diabetes, obesity and vascular complications, 3 major groups of miRNAs can be identified: inflammation-associated miRNAs (inflammaMiRs), angiogenesis-associated miRNAs (angioMiRs) and senescence-associated miRNAs (seneMiRs) [89] (Figure 2).

Overexpression of proinflammatory miR-155 in ECs has been linked to inhibition of cell proliferation/migration and subsequent impaired re-endothelialisation [90]. The miR-155-mediated disruption of gap- and adherents-junctions perturbs the endothelial monolayer barrier, resulting in increased permeability and enhanced macrophages infiltration that may facilitate atherosclerotic plaque formation [90] and progression [91]. Interestingly, a 5-fold increase for both miR-155 and miR-146a has been observed in kidney samples from patients with diabetic nephropathy; the close correlation between increased miR-155 and creatinine levels suggests the possibility to consider these miRNAs as useful biomarkers in the evaluation of the disease progression [89]. Additional support to the role of miRNAs as biomarkers comes from experimental studies: a gradual increase of both miR-155 and miR-146a has been observed during progression of T1DM and T2DM in diabetic nephropathy rats [92]; moreover, exposure to high glucose enhances the expression of miR-155 and miR-146a, concomitantly increasing levels of TNFα, TGFβ1 and NF-kB responsible for inflammatory lesions in human renal glomerular endothelial cells (HRGEC) [92]. Increased levels of miR-146a seem also to correlate with significantly higher risk of ischemic stroke under hyperglycaemic conditions and single nucleotide polymorphisms in MIR146A gene may contribute to enhance disease susceptibility [93]. 

In contrast with other inflammaMiRs, whose levels increase proportionally to the glucose exposure, miR-126 is highly expressed in ECs under basal conditions and exerts a protective role in controlling inflammation as well as in regulating cell migration and survival [94,95]. miR-126 modulates vascular inflammation through inhibition of VCAM1 expression, usually involved in the adhesion of leukocytes to ECs [96]. Consistent with this, inhibition of miR-126 correlates with increased activation of NF-kB pathway, that promotes the expression of pro-inflammatory cytokine TNFα and adhesion molecule VCAM1, with subsequent enhanced adhesion of leukocytes to endothelium [96]. In addition, miR-126 modulates angiogenesis and vascular integrity via its inhibitory activity on VEGF signalling [97]. A significant reduction of miR-126 has been measured in plasma as well as in apoptotic ECs from pre-diabetic patients some years before the onset of the disease, highlighting its potential role as an early predictive biomarker for diabetic vascular complications [98,99]. Indeed, miR-126 appears to regulate proliferation and migration of bone marrow-derived endothelial progenitor cells (EPCs), whose circulating levels and regenerative vascular ability is significantly reduced in diabetic patients [19,100,101,102,103]. The association between impairment of EPCs functioning and downregulation of miR-126, together with miR-130a, miR-21 and miR-27a/b observed in samples from T2DM patients [104] supports the idea that variations of miR-126 levels might be indicative of disease progression. In line with this, miR-126 levels have been found significantly increased both in patients with pre-diabetic syndrome and T2DM after six months of treatment with diet and exercise, alone or in combination with insulin, respectively [105]. 

Lately, in human ECs, a positive correlation has been observed for a group of 10 miRNAs (miR-26a-5p, -26b-5p, -49b-3p, -29c-3p, -125b-1-3p, -130b-3p, -140-5p and -221-3p and -320a) whose levels gradually increase when cells are exposed to increasing glucose concentrations. For all these miRNAs a crucial role in endothelial dysfunction has been suggested and for seven of them the association with endothelial cells apoptosis has been proposed [106].

Senescence-associated miRNAs could potentially represent additional risk biomarkers of diabetes-associated vascular complications, based on the notion that a prolonged exposure to oxidative stress accelerates the endothelial ageing and significantly contributes to the evolution of cardiovascular diseases. Some miRNAs are known to counteract oxidative stress-induced senescence; for example, the main mechanism by which miR-146a regulates senescence is controlling the expression of NOX4, the key isoform of the NADPH-complex that catalyses the reduction of molecular oxygen to ROS in ECs [107]. As expected, in T2DM patients, Mir146-a is downregulated in the peripheral blood mononuclear cells (PBMC) and its plasma levels are reduced under insulin resistance, poor glycaemic control and high pro-inflammatory cytokine levels [108]. Thus, enhancement of miR-146a expression with subsequent reduction of NOX4 synthesis/activity might represent a strategy to reduce the oxidative stress and associated senescence in human ECs.

In turn, ROS may induce the increase of several miRNAs, including miR-200c and other miR-200 family members, termed oxidative stress-responsive miRNAs. In ECs the overexpression of miR-200c is responsible for growth arrest, apoptosis and senescence [109]. Increased levels of miR-200c disrupt the regulatory loop between Sirtuin 1 (SIRT1), Forkhead box O1 (FOXO1) and eNOS, three proteins functionally related that modulate EC function and NO/ROS production, playing an important role in vascular homeostasis [110]. Sirtuin1 is a NAD+-dependent class III histone deacetylase with anti-oxidant and anti-inflammatory effects that prevents endothelial senescence and promotes the bioavailability of NO. Both supraphysiological ROS levels and ageing decrease the expression/activity of SIRT1, promoting endothelial dysfunction by impaired eNOS expression and/or NO bioavailability, as well as proatherogenic events and senescence [111,112,113]. As a direct target of SIRT1 deacetylation, FOXO1 activates the transcription on both SIRT1 and ROS scavengers such as catalase and SOD2. In the context of diabetes, the aberrant overexpression of miR-200c causes down-modulation of SIRT1, inhibits the transcription of FOXO1, increases the phosphorylation of p66Shc protein on Ser-36 and inhibits ROS scavenger expression/activity, thus contributing to enhance the oxidative stress as well as the formation of atherosclerotic plaque [110]. Several data show that a complex network of chromatin remodellers regulate p66Shc transcription by inducing both demethylation and acetylation of H3K9 [114]. Conversely, in vivo gene silencing of p66Shc restores endothelial insulin response acting on IRS-1/Akt/eNOS and NF-kB pathway [115]. These modifications in p66Shc gene promoter may lead to the pathogenesis of endothelial insulin resistance, increasing vascular risk in the context of diabetes as well as obesity [114].

At present, few data are available on the role of lncRNAs in diabetes-related endothelial dysfunction. However, lncRNAs are regarded as major players in damaging pancreatic β-cells in both T1DM and T2DM. In human ECs, approximately 100 lncRNAs were found upregulated and 186 down-regulated [116] upon exposure to high glucose. Moreover, the aberrant expression of circulating lncRNAs in T2DM patients seems mostly related to processes such as inflammation, immune response, insulin resistance and regulation of insulin secretion [117]. Recently, the lncRNA MALAT1 (metastasis associated lung adenocarcinoma transcript 1) has been found increased in ECs exposed to high glucose, suggesting its potential role in upregulating inflammatory mediators underlying micro- and macrovascular complications [118]. Although the majority of lncRNAs enhances endothelial dysfunction in diabetes, some of them may have a protective function: for example, the lncRNA-MEG3 facilitates the activation of PI3K/AKT signalling in ECs [119]; accordingly, its expression is down-regulated in retinal vessel of STZ-induced diabetic mice and in ECs exposed to hyperglycaemic and oxidative stress conditions, while MEG3 knockdown associates with capillary degeneration and microvascular leakage, worsening retinal vessel dysfunction in diabetic mice [119].

## 4. Epigenetic Mechanisms as Potential Therapeutic Targets in T2DM Endothelial Dysfunction

Advances in understanding the mechanisms involved in reversible epigenetic changes encourages the possibility to develop new therapeutic interventions that can delay the harmful effects of diabetes on endothelial function. For the majority of these strategies, their possible use in clinical practice is still far from being achieved but might represent an additional opportunity in combination with standard anti-diabetic treatments. The following paragraphs summarize only few examples of the most promising and fascinating findings up to date.

### 4.1. Endothelial Progenitor Cells (EPCs)

The emergence of a “glycaemic memory” suggests the need to develop an early treatment with drugs reducing ROS activities that may improve the control of metabolism and ultimately minimize the hyperglycaemia-related long-term vascular complications [21,27]. The growing evidence that EPCs are localized, in a limited amount, in the vascular wall [120,121] suggests that this cellular reservoir may serve to replace a dysfunctional endothelium, even in the earliest stages of the atherogenic process [122]. Thus, EPCs have been regarded both as potential biomarkers to identify the onset and progression of vascular disease and a future cell-based therapeutic strategy to help vascular regeneration of injured vessels [123]. However, diabetic EPCs show not only altered proliferation and adhesion but also reduced ability to be incorporated into vascular structures [124]. One explanation hypothesizes that EPCs could also represent a potential carrier of endothelial glycaemic memory in a diabetic context and therefore infusion of autologous EPCs in a diabetic patient after in vitro expansion may not be the most promising solution [19,125]. In the attempt to overcome this issue and reprogram glycaemic memory, some studies have evaluated the effects of tricostatin A (TSA), a histone inhibitor of class I and II deacetylases (HDAC1/2) [126]. HDAC1 is known to play a key role in inhibiting differentiation and arresting EC growth and its upregulation in ECs is associated with reduced eNOS activity and decreased NO production [127]. Treatment of endothelial colony forming cells (ECFCs) with TSA in vitro has been effective in increasing the efficiency of revascularization [128], as well as in upregulating eNOS mRNA levels in VSMC [129]. Similarly, the combined treatment with TSA and DZNep, a global histone methylation inhibitor, increases eNOS expression in EPC through the simultaneous decrease of the repressive histone H3K27me3 signature and increase of its acetylation to the proximal eNOS promoter [43,130]. Furthermore, ex vivo treatment of murine EPCs with anti-miR-15a/16 resulted in increased VEGFAb and Akt-3 levels associated with improved recovery after ischemia limb, indicating a potential therapeutic strategy to enhance EPC activity before autologous transplantation [131]. Unfortunately, the lack of an extensive study of EPCs epigenome in diabetic organisms and the high toxicity of the drugs make their clinical use limited to selected patients nowadays [132,133].

### 4.2. Histone Acetyl-Transferases (HAT) and Histone Deacetylases (HDAC)

Targeting HATs to prevent endothelial diabetic dysfunction is still far from being achieved. However, increased HAT activity (i.e., histone H4 acetylation and histone H3 phospho-acetylation) by shear stress promotes hematopoietic stem cells differentiation towards the endothelial lineage [134]. In ECs, shear stress stimulates p300-mediated acetylation of both the NFkB p65 subunit as well as the eNOS isoform [135]. Consistent with these findings, p300 knockdown decreases NFkB as well as AP1 and CREB transcription factor expressions [136] and HDAC3 counteracts p300 function through NFkB deacetylation [127]. This last observation, together with the finding that aberrant recruitment and HDAC overexpression is associated with several pathological conditions including diabetes [137,138,139,140,141], suggests that targeted HDAC3 inhibition could be a potential strategic target to increase eNOS expression for the treatment of diabetes-associated vascular disease.

Indeed, the inhibition or RNAi-mediated knockdown of HDAC3 has been shown to enhance acetylation and subsequent activation of the peroxisome proliferator-activated receptor gamma (PPARγ) in the absence of an exogenous ligand [142]. This correlates with increased expression of PPARγ target genes, improvement of insulin signalling and enhanced eNOS activation in ECs [142]. Thus, acetylation of PPARγ may represent a ligand-independent mechanism of PPARγ activation and the selective inhibition of HDAC3 might greatly contribute to reinforce the effects of the synthetic PPARγ ligands thiazolidinediones [143].

Among HDAC inhibitors, sodium butyrate appears to have a protective role in experimental models of myocardial infarction and atherosclerosis [144]. Curcumin (curcuma longa) has shown to prevent vascular dysfunction by reducing the acetylation of NF-kB in microvascular ECs from diabetic rodents [145,146] and improve proteinuria while reducing pro-fibrotic cytokines such as TGFβ and IL8 in diabetic patients [147]. Despite some potential beneficial effects, however, the use of non-specific first-generation HDAC inhibitors and the high structural similarity between HDAC1, 2 and 3 represents one of the main obstacles in the development of HDAC3 selective drugs [143].

### 4.3. DNA Methylation and Histone Modifications

Histone hypomethylation of H3K4 contributes to down-regulated gene expression of SOD2 [78,148]. Reduced levels of H3K4me1 and H3K4me2 are observed at the SOD2 gene promoter under dysglycaemic conditions [78] and high glucose is known to increase H3K4me1 and reduce the expression levels of H3K4me2 and H3K4me3 to the NFkB promoter in human ECs [149]. Furthermore, the histone codes H3K9ac, H3K12ac, H3K4me2 and H3K4me3 suppress the transcription of eNOS and impair NO production [129]. The enzyme Set7, that methylates lysine residues of both histone and non-histone proteins, is overexpressed in HAEC exposed to high glucose and seems directly involved in activation of numerous pro-inflammatory genes even after the restoration of normoglycaemic conditions [70,74]. Indeed, Set7 inhibition in monocytes results in limited NFkB signalling, with concomitant reduction of the pro-inflammatory potential and monocytic adhesion to vascular ECs [73]. Experimental inhibition of Set7/NF-kB inflammatory signalling has obtained with Quercus infectoria in bone marrow-derived macrophages exposed to a diabetic environment [150]. Histone H2AK119 mono-ubiquitination (H2AK119-Ub) has a key role in regulating Set7 activity; accordingly, lower levels of Set7 and prevention of renal fibrosis have been correlated to increased protein expression of H2AK119-Ub in response to aspirin in glomeruli from diabetic animals [151]. Although very preliminary, these observations will hopefully contribute to add new options to the list of therapeutic targets for diabetes vascular complications.

### 4.4. Non Coding RNAs

Among epigenetic markers of potential therapeutic use, this is certainly the most advanced field. The miRNA-based therapeutics includes two main approaches: miRNA inhibition therapy (miRNA inhibitors), which involves down-modulation of aberrantly over-expressed miRNAs using either complementary antisense oligonucleotides or miRNA sponges and miRNA replacement therapy, based on restoration of down-regulated miRNA activity, using synthetic miRNA mimics [152].

Locked Nucleic Acid (LNA)-modified anti-miRNAs, called antagomiRs, possess high thermal stability when hybridized with their target mRNAs and high sensitivity and specificity in detecting the targeted miRNAs [153,154]. Several antagomiRs have been approved by the FDA and many others are in different clinical phases for the treatment of different pathological conditions [155].

In the mechanisms responsible for the loss of endothelial repair capacity, the role of miR-483-3p has emerged [156] due to its high expression in macrophages and in aortic cells of diabetic subjects compared to controls. The anti-miR-483-3p-LNA improves endothelial regenerative capacity in vivo, supporting the promising therapeutic potential of miR-483-3p inhibitors for the treatment of vasculopathies in diabetic patients [157].

Transient hyperglycaemia is known to increase miR-23b-3p expression, targeting and inhibiting SIRT1 mRNA in ECs [158]. As a consequence, the increase in NF-kB p65 acetylation establishes a pro-inflammatory and pro-oxidant state. In turn, a reduced expression of miR-23b-3p increases deacetylated NF-kB levels by maintaining SIRT1 expression in retinal ECs [158]. Under physiological conditions, SIRT1 deacetylation stops the transcription of p53 dependent genes [159] [160], including miR34-a, known to directly target the cytoplasmic SIRT1 mRNA and inhibiting SIRT1 protein production [161,162]. A dysregulation of the SIRT1/miR34a/p53 axis in diabetes has been proposed based on high levels of acetylated p53 and concomitant drastic decrease of SIRT1 levels in ECs [163]. Inhibition of p53 and miR-34a attenuates the high glucose-induced endothelial inflammation and oxidative stress by increasing SIRT1 levels: in addition, LNA-modified antimiR-34 family seem able to improve systolic function [164], suggesting its potential use in diabetic cardiovascular complications. 

On the other hand, under high glucose-induced vascular EC dysfunction, the expression of other miRNAs is substantially decreased: for example, reduced levels of miR-106 have been associated with increased expression of HMGB1 (High mobility group box 1), a nuclear DNA-binding protein released from necrotic cells, monocytes/macrophages and endothelial cells [165,166]. The use of miR-106 mimics significantly reduced the high glucose-induced apoptotic mechanisms, suggesting the protective role of miR-106 on endothelial function [166,167].

Among miRNAs showing a protective potential on endothelial activity, miR-142 transfection has been capable to increase the gene expression of VEGFR2, PI3K, Akt and eNOS, with consequent production of NO in EPCs [168], by down-regulating mRNA expression of ADAMTS-1 (a disintegrin and metalloproteinase with thrombospondin motif-1). Low levels of miR-126 in diabetic EC are associated with decreased proliferation, migration and NO production, through suppression of the PI3K/AKT/eNOS pathway [104,169].

MiR-342-3p, an obesity-associated miRNA, has recently been suggested to act as pro-angiogenic factor and is down-modulated in ECs from T2DM mice models and human diabetic patients, contributing to aggravate endothelial dysfunction by slowing down proliferation and endothelial migration [170]. MiR-342-3p directly targets the 3′UTR of IGF-1R (insulin-like growth factor receptor) acting as a potent tumour suppressor in hepatocellular carcinoma through inhibition of IGF-1R-mediated PI3K/Akt/GLUT1 signalling pathway [171]. Low levels of miR-342-3p are detected in ECs under diabetes; since the IGF-1R is structurally similar to IR, the continuous insulin binding to the IGF-1R could lead to desensitization of the insulin pathway at the level of vascular endothelium, altering cell proliferation and triggering an inflammatory environment [170].

LncRNAs could also find a place as new biomarkers for the early diagnosis and prediction of diabetes-induced micro-vascular complications. For instance, MEG3 down regulation, associated with increased vascular injury and hyperglycaemia-induced inflammation, might be useful for the identification of a new therapeutic approach in the treatment of vascular complications that arise in diabetic individuals [172]. Moreover, ncRNAs have shown high stability in exosomes and organic biofluids (urine, serum and plasma) [173]. This, together with the availability of experimental procedure for their detection and quantification [174], makes them eligible of becoming non-invasive biomarkers for the early diagnosis of diabetes-associated vascular complications. In this regard evidence is accumulating regarding the role played by exosomes and their bioactive content in the pathophysiology of this disease (see Reference [175] for comprehensive review). Exosomes are small extracellular vesicles that shuttle proteins, miRNAs and other lncRNAs, shielding these molecules from enzymatic degradation and ultimately deliver them to recipient cells, both within the tissues and throughout the entire circulatory system. Pro-survival and angiogenesis assays in ECs have shown that exosomes contribute to regulate vascular survival and integrity and circulating exosomes isolated from patients with metabolic syndrome have been able to induce vascular dysfunction in control mice [176]. Moreover, levels of both miR-146 and miR-126 are deregulated in exosome from diabetic patients and may contribute to altered IRS-1 expression and vascular integrity in these subjects. Recently, exosomes isolated from the plasma of diabetic subjects or db/db diabetic mice were shown to be enriched in arginase 1 (ARG1), which could be transferred to ECs in vitro and in vivo and play a role in decreased NO production and impaired vascular function [177]. At present, the partial knowledge of the internal content of exosomal vesicles and the heterogeneity of extracellular circulating RNAs (exRNA) [178] requires caution [179,180] but it is conceivable that exosomes may represent suitable delivery vehicles for signalling molecules and targeted drugs in diabetes. 

## 5. Epigenetic Modifications Induced by Standard Anti-Hyperglycaemic Drugs

T2DM patients represent a heterogeneous group of subjects in which individual features, specific genetic background, disease progression and associated co-morbidities may significantly modify the effectiveness of anti-diabetic therapy. The attempt to personalize the diabetes treatment as much as possible is currently based on the growing knowledge of drug mechanism of action and genetic-driven pharmacokinetics differences: for example, glucagon-peptide-1 receptor agonists (GLP-1 RA) will not be successful in subjects with severe insulin deficiency and thiazolidinediones (TZD) work better in insulin-resistant obese patients than in normal-weight patients [181,182]. Sulfonylureas (SU) in the treatment of monogenic form of diabetes (MODY, maturity-onset diabetes of the young) [183] appear to work particularly well in patients carrying a MODY3 HNF1A (hepatocyte nuclear factor 1 homeobox A) [184] and MODY1 HNF4A mutations [185]. A reduced tolerance to metformin (Met) has been detected in patients carrying variations in the organic cation transporter 1 (OCT1) responsible for intestinal Met absorption [186,187]. Not all therapeutic strategies for T2DM protect or reverse endothelial dysregulation with the same efficacy and data regarding the ability of anti-diabetic drugs to attenuate the “metabolic memory” through epigenetic mechanisms are still very limited. Nevertheless, a growing number of experimental and clinical studies have ascertained the specific effects on endothelial function and inflammatory signalling for most of the drugs used [188]. 

For Met, a positive protective activity in prevention of endothelial senescence [189] has been suggested by the increased SIRT1 expression and activity with subsequent reduction of aging and oxidative stress in ECs [190,191], together with the increased amount of circulating EPCs in diabetic patients [192]. More recently, Met treatment has been shown to restore the endothelial expression of miRNAs of the let-7 family [193] whose hypothetical role is to protect against atherosclerotic plaques [194] and whose levels are reduced in diabetic patients [193]. Concomitantly, levels of miR-221 and miR-222, that act by promoting intima thickening and reducing eNOS expression [166,195,196], have been found lower in patients treated with Met when compared to subjects receiving other therapies [197].

Activation of GLP-1R improves endothelial function in high risk cardiac patients [198] and treatment with GLP-1RA stimulates proliferation via eNOS-, PKA- and PI3K/Akt-dependent pathways in endothelial cells from human coronary arteries [199]. The anti-inflammatory effects of GLP-1RA liraglutide -via NF-kB downregulation and reduced synthesis of inflammatory cytokines (MCP-1, TNFα, INF-γ and IL-6)- and the concomitant eNOS activation suggest its protective vascular effects, irrespective of glucose-lowering activity [200]. Indeed, liraglutide beneficial effects on vascular function have been linked to downregulated expression of 12 miRNAs including miR-93-5p, miR-181a-5p and miR-34a-5p and upregulated levels of 33 miRNAs including miR-26a-5p [201]. 

Dipeptydil-peptidase-4 (DPP-4) inhibitors (gliptins) slow down the endogenous degradation of GLP-1. EC are the main source of soluble DPP-4 enzymes, whose expression and activity may increase after chronic exposure to high doses of glucose [202]. At present, the clinical evidence of a protective vascular effect of DPP-4 inhibitors is uncertain but experimental studies support the idea that some of these drugs may improve endothelial function and decrease blood pressure [203,204]. In vitro, anagliptin appears to counteract hyperglycaemia-induced endothelial dysfunction via down regulation of NLRP3 inflammasome [205], whose activation increases the release of HMGB1 (High Mobility group box protein-1) and promotes RAGE-mediated pathways [206]. 

The potential use of inhibitors of the nuclear enzyme poly (ADP ribose) polymerase (PARP) as a therapeutic strategy in diabetic vascular complications [207] has been suggested following the observation that PARP-1 is overactivated in diabetic endothelial dysfunction [208,209] and associated to downregulation of endothelial NADPH activities and increased ROS formation [210]. At present, clinical therapy with PARP inhibitors is far from being achieved; however, a shorter-term indirect approach may involve the α-lipoic acid, which has been demonstrated to inhibit diabetes-induced PARP overactivation in preclinical studies [21,211]. Met has also been shown to suppress endothelial PARP activation in vitro but the underlying molecular mechanisms are not fully known [212]. 

## 6. Conclusions

The current approaches employed for monitoring plasma glucose levels are not suitable for quantifying the degree of endothelial dysfunction [213] and available biomarkers of endothelial dysfunction might only be of some use in late stages of diabetic complications [214]. The increasing understanding of the epigenetic machinery underlying diabetic endothelial dysfunction might significantly contribute to the field of precision medicine, providing tools to identify individuals at high risk of developing long-term macro and micro-vascular complications, as well as diabetic individuals eligible to receive novel treatments which, in association with traditional anti-diabetic drugs, will hopefully reach clinical practice in the future. Furthermore, in a truly personalized approach to management and health care of diabetic patients, the contribution of pharmacogenomics and pharmacogenetics will be not less significant as providing information on the drug treatment with the highest efficacy and least toxicity in individual subjects. 

At present, mechanisms that may link epigenetic changes to diabetic endothelial dysfunction in a cause-effect relationship are far from being unequivocally clarified. Importantly, epigenetic changes may be species-specific and this possibility should be carefully considered when translating the epigenetic results from animals to humans. Moreover, translation of epigenetic strategies into the clinical setting is currently complicated by several reasons as, for example, the mRNA-lncRNA interactions in the vasculature, the ability of a single ncRNA to regulate different biological processes, the high toxicity of experimental molecules. Nevertheless, some encouraging aspects and potential advantages may also be considered: ncRNAs can be easily detected in body fluids, they maintain high plasma stability and laboratory techniques widely used may be employed in their evaluation. 

Thus, despite current uncertainties and limitations, the relentless research aimed at identifying specific epigenetic mechanisms related to endothelial dysfunction in diabetic patients and pre-diabetic subjects will surely provide important tools in predicting, diagnosing and monitoring treatment efficacy of this complex multifactorial disease.

## Abbreviations

T2DM	Type 2 Diabetes Mellitus
eNOS	Endothelial NO Synthase
GWAS	Genome Wide Association Studies
AMPK	5’AMP-activated protein kinase
BH4	Tetrahydrobiopterin
ROS	Radical Oxygen Species
NO	Nitric Oxide
AGEs	Advanced Glycosylated End products
mtDNA	Mitochondrial DNA
SNP	Single nucleotide polymorphism
EWAS	Epigenome-wide association studies
TSS	Transcription Start Site
TF	Transcription Factor
DNMT	DNA-methyltransferase
MeCpG	methylated CpG Binding-Protein
HDAC	Histone deacetylase
HMT	Histone methyltransferase
HAT	Histone acetyltransferase
KMT	Lysine methyltransferase
ncRNA	Non-coding RNA
rRNA	Ribosomal RNA
tRNA	Tranfer RNA
snRNA	small nuclear RNA
snoRNA	small nucleolar RNA
siRNAs	small interfering RNA
piRNAs	piwi-associated RNA
miRNA, miRs	micro RNA
lncRNA	Long non-coding RNA
hs-CRP	high-sensitive C-reactive protein
AHBA	alpha-hydroxybutyric acid
LGPC	linoleonylglycerophosphorcholine
ECs	endothelial cells
PTHM	Post-translational histone modifications
HAEC	human aortic endothelial cells
MMPs	matrix metalloproteinases
LINE-1	Long Intersperted Nuclear Element 1
HRGEC	human renal glomerular endothelial cells
EPCs	endothelial progenitor cells
PBMC	peripheral blood mononuclear cells
SIRT1	Sirtuin 1
FOXO1	Forkhead box O1
TSA	Tricostatin A
ECFCs	Endothelial Colony Forming Cells
HMGB1	High mobility group box 1)
TZD	thiazolidinediones
SU	Sulfonylureas
MODY	Maturity-Onset Diabetes of the Young
HNF1A	hepatocyte nuclear factor 1 homeobox A
Met	Metformin
OCT1	Organic Cation Transporter 1
PARP	Poly (ADP ribose) Polymerase
